# Noise-induced hearing loss increases the temporal precision of complex envelope coding by auditory-nerve fibers

**DOI:** 10.3389/fnsys.2014.00020

**Published:** 2014-02-17

**Authors:** Kenneth S. Henry, Sushrut Kale, Michael G. Heinz

**Affiliations:** ^1^Department of Speech, Language, and Hearing Sciences, Purdue UniversityWest Lafayette, IN, USA; ^2^Weldon School of Biomedical Engineering, Purdue UniversityWest Lafayette, IN, USA

**Keywords:** amplitude modulation, auditory nerve, sensorineural hearing loss, temporal envelope, temporal resolution, Wiener-kernel analysis

## Abstract

While changes in cochlear frequency tuning are thought to play an important role in the perceptual difficulties of people with sensorineural hearing loss (SNHL), the possible role of temporal processing deficits remains less clear. Our knowledge of temporal envelope coding in the impaired cochlea is limited to two studies that examined auditory-nerve fiber responses to narrowband amplitude modulated stimuli. In the present study, we used Wiener-kernel analyses of auditory-nerve fiber responses to broadband Gaussian noise in anesthetized chinchillas to quantify changes in temporal envelope coding with noise-induced SNHL. Temporal modulation transfer functions (TMTFs) and temporal windows of sensitivity to acoustic stimulation were computed from 2nd-order Wiener kernels and analyzed to estimate the temporal precision, amplitude, and latency of envelope coding. Noise overexposure was associated with slower (less negative) TMTF roll-off with increasing modulation frequency and reduced temporal window duration. The results show that at equal stimulus sensation level, SNHL increases the temporal precision of envelope coding by 20–30%. Furthermore, SNHL increased the amplitude of envelope coding by 50% in fibers with CFs from 1–2 kHz and decreased mean response latency by 0.4 ms. While a previous study of envelope coding demonstrated a similar increase in response amplitude, the present study is the first to show enhanced temporal precision. This new finding may relate to the use of a more complex stimulus with broad frequency bandwidth and a dynamic temporal envelope. Exaggerated neural coding of fast envelope modulations may contribute to perceptual difficulties in people with SNHL by acting as a distraction from more relevant acoustic cues, especially in fluctuating background noise. Finally, the results underscore the value of studying sensory systems with more natural, real-world stimuli.

## Introduction

People with sensorineural hearing loss (SNHL) commonly have difficulty understanding speech under real-world listening conditions, even with amplification from a modern hearing aid (Duquesnoy, 1983; Woods et al., 2010). Research conducted over the last several decades has uncovered changes in cochlear frequency tuning with SNHL that most likely contribute to speech perception problems in these listeners. While the normal-hearing cochlea decomposes broadband signals like speech into a number of sharply tuned “auditory filter” channels for central processing, SNHL causes an increase in the bandwidth of auditory filters that consequently decreases resolution of spectral features by the cochlea (Young, 2012). Furthermore, SNHL causes downward shifts in the best frequency of tuning, particularly in the base of the cochlea, which are also likely to contribute to perceptual impairment (Liberman, 1984; Henry and Heinz, 2013).

In addition to diminished spectral resolution, perceptual difficulties in people with SNHL might also reflect changes in auditory sensitivity to the temporal structure of sound. The effects of SNHL on sensitivity to temporal fine structure and slower varying temporal envelope cues are both topics of active debate and investigation (Lorenzi et al., 2006; Hopkins et al., 2008; Swaminathan and Heinz, 2012). In the current report, we focus on temporal envelope sensitivity. While several studies suggest that SNHL might cause an increase in the perceptual salience of temporal envelope structure that could adversely affect speech perception in fluctuating background noise (through loudness recruitment; Moore and Glasberg, 1993; Moore et al., 1995, 1996), other studies suggest that envelope sensitivity is relatively unaffected by SNHL (Bacon and Gleitman, 1992; Moore et al., 1992; Lorenzi et al., 2006) or even diminished (Bacon and Viemeister, 1985; Formby, 1987; Grant et al., 1998). Physiological data from non-human animals have the potential to clarify changes in temporal envelope sensitivity with SNHL.

Our current physiological knowledge of temporal envelope coding in the impaired cochlea is limited (e.g., Kale and Heinz, 2010, 2012). Consistent with enhancement of envelope coding, these studies showed that noise-induced SNHL amplifies phase locking to the temporal envelope of sinusoidally amplitude modulated (SAM) tones and single-formant stimuli in auditory-nerve fibers of anesthetized chinchillas (Kale and Heinz, 2010). Amplified envelope coding was observed over a wide range of modulation frequencies and did not appear to alter the shape of temporal modulation transfer functions (TMTFs) plotting response amplitude as a function of modulation frequency (Kale and Heinz, 2012). Hence, it appears from the SAM-tone data that SNHL may amplify the neural representation of envelope structure in the cochlea without altering the precision with which temporal modulations are encoded, i.e., without extending modulation coding to higher modulation frequencies.

**Figure 1:**
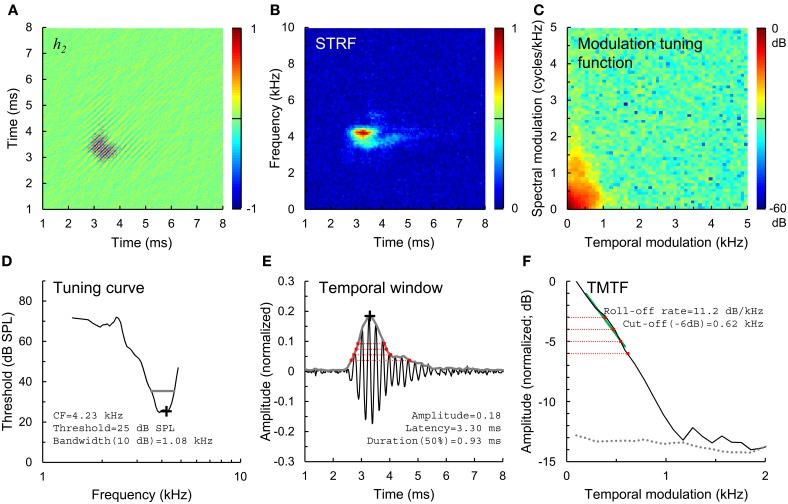
**Estimation of temporal envelope coding. (A)** 2nd-order Wiener kernel (*h*_2_), computed from 2nd-order cross correlation of a broadband Gaussian noise stimulus and spike train response of a normal-hearing auditory-nerve fiber. Time axes (**A,B,E**) indicate time relative to the occurrence of a spike, and are plotted from a lower limit of 1 ms rather than 0 ms to more clearly show the structure of the kernels. Normalized amplitude color scales **(A–C)** are drawn to the right of each panel. **(B)** Spectro-temporal receptive field (STRF), or mean stimulus spectrogram preceding a spike, calculated from *h*_2_. **(C)** Modulation tuning function, calculated from the STRF. **(D)** Tuning curve showing threshold and CF (black cross) and the 10-dB bandwidth of frequency tuning (gray line). **(E)** Temporal window of sensitivity (gray line), computed as the amplitude envelope of the 1st eigenvector of *h*_2_. Temporal windows were used to calculate the amplitude and latency of envelope coding (black cross) and duration of temporal sensitivity at 50, 40, 30, and 20% of peak amplitude (red dotted lines). **(F)** Temporal modulation transfer function (TMTF, black line), calculated from the modulation tuning function, and TMTF noise floor (gray dotted line). TMTFs were characterized based on roll-off rate (green line) and cut-off modulation frequencies measured 3, 4, 5, and 6 dB down from peak amplitude (red dotted lines).

In the present study, we extend this previous physiological work by studying temporal envelope coding using more complex, Gaussian noise stimuli with broad frequency bandwidth and a dynamic temporal envelope. Wiener-kernel analyses of auditory-nerve fiber responses were used to quantify the preferred spectral and temporal stimulus features driving the neuron (van Dijk et al., 1994; Lewis et al., 2002; Recio-Spinoso et al., 2005; Temchin et al., 2005). The 2nd-order Wiener kernel (*h*_2_; Figure 1A) is a time domain representation of the spectro-temporal receptive field (STRF; Figure 1B) or mean spectrogram in the 10–20 ms time window preceding a spike (the STRF is the 1-dimenstional Fourier transform of *h*_2_; Lewis and van Dijk, 2004). Just as STRFs that are compact in frequency indicate sharp frequency tuning (i.e., spikes are driven by a narrow range of acoustic frequencies), STRFs and *h*_2_ that are compact in time indicate high temporal precision, i.e., spikes are driven by acoustic energy falling in a short temporal window.

In previous studies, we used Wiener kernel analyses to quantify the effects of noise-induced SNHL on the frequency tuning of phase-locked responses to the temporal fine structure and envelope of broadband Gaussian noise stimuli (e.g., Henry and Heinz, 2013). Here, we quantify the effects of SNHL on the temporal precision, amplitude, and latency of envelope coding at the level of the auditory nerve in anesthetized chinchillas. The results show that at equal stimulus sensation level, noise-induced SNHL increases the temporal precision of envelope coding by 20–30%. Furthermore, SNHL is associated with a decrease in response latency of 0.4 ms and amplification (~50%) of envelope coding in fibers with CFs from 1–2 kHz.

## Materials and methods

### Animals

All procedures were performed in chinchillas and approved by the Purdue Animal Care and Use Committee. The neurophysiological data presented here were collected from 10 normal hearing control animals (143 fibers), 6 animals exposed to a 50 Hz band of Gaussian noise with a center frequency of 2 kHz for 4 h at 115 dB SPL (76 fibers), and 5 animals exposed to an octave band of Gaussian noise with a center frequency of 500 Hz for 2 h at 116 dB SPL (46 fibers).

### Noise overexposures

Noise overexposures were performed in a sound-attenuating booth under anesthesia using either a pair of dynamic loudspeakers (Fostex FT28D; for 2 kHz exposures) or single enclosed woofer (Selenium 10PW3; for 500 Hz exposures) suspended 25–30 cm above the animal. Anesthesia was induced with xylazine (1–2 mg/kg subcutaneous) followed after several minutes by ketamine (50–65 mg/kg intraperitoneal). Atropine (0.05 mg/kg intramuscular) was given to control mucous secretions and eye ointment was applied. Animals were held in position with a stereotaxic device, and body temperature was maintained at 37°C using a feedback controlled heating pad (Physitemp TCAT2LV or Harvard Apparatus 50–7220F). Supplemental injections of ketamine (20–30 mg/kg intraperitoneal) were given as needed to maintain an areflexic state.

### Neurophysiological recordings

Neurophysiological data were recorded from auditory-nerve fibers under anesthesia 3 or more weeks after the noise overexposure using standard procedures in our lab (e.g., Kale and Heinz, 2010, 2012). Anesthesia was induced with xylazine and ketamine as described above, but maintained with sodium pentobarbital (~15 mg/kg/2 h intravenous) for neurophysiological recordings. Physiological saline (1–2 ml/2 h intravenous) and lactated ringers (20–30 ml/24 h subcutaneous) were also given, and a tracheotomy performed to facilitate breathing. Animals were positioned in a stereotaxic device in a sound-attenuating booth. The skin and muscles overlying the skull were transected to expose the ear canals and bullae, and both ear canals were dissected to allow insertion of hollow ear bars. The right bulla was vented through 30 cm of polyethylene tubing. A craniotomy was opened in the posterior fossa and the cerebellum partially aspirated and retracted medially to expose the trunk of the auditory nerve bundle. Acoustic stimuli were presented through the right ear bar with a dynamic loudspeaker (Beyerdynamic DT48) and calibrated using a probe microphone placed within a few mm of the tympanum (Etymotic ER7C). Neurophysiological recordings were made using a 10–30 MΩ glass microelectrode advanced into the auditory nerve with a hydraulic microdrive (Kopf 640). Recordings were amplified (Dagan 2400A) and band-pass filtered from 0.03 to 6 kHz (Krohn-Hite 3550). Spikes were identified using a time-amplitude window discriminator (BAK Electronics) and timed with 10-μs resolution.

**Figure 2:**
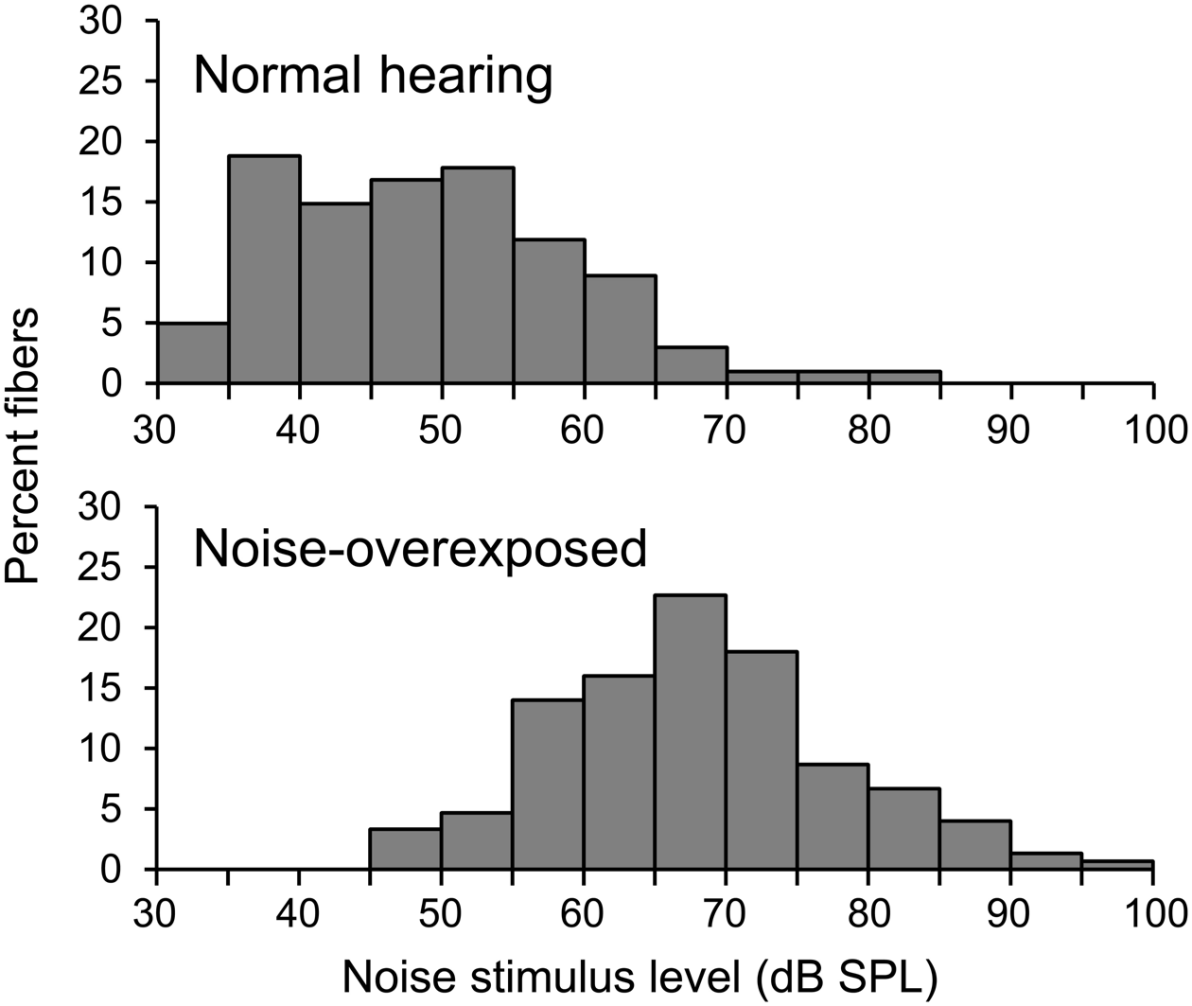
**Noise stimulus level**. Histograms showing the distribution of noise stimulus levels presented to normal-hearing and noise-overexposed fibers.

Single fibers were isolated by listening for spikes on a monitor speaker while advancing the electrode through the auditory nerve during periodic stimulation with broadband noise. When a fiber was encountered, a tuning curve was recorded (Figure 1D, black line) using an automated procedure that tracked, as a function of stimulus frequency, the minimum SPL of a 50-ms tone required to evoke at least 1 more spike than a subsequent 50-ms silent period (Chintanpalli and Heinz, 2007). CF (Figure 1D, cross) was identified as the frequency of best sensitivity or, in noise-overexposed fibers, as the frequency of the breakpoint in the high frequency slope of the tuning curve because this value provides a robust estimate of CF prior to cochlear damage (Liberman, 1984). The bandwidth of the tuning curve 10 dB above threshold was also quantified (Figure 1D, gray line). Next, a sequence of 9 broadband Gaussian noise stimuli were presented repeatedly for up to 10 mins at 10–15 dB above the threshold for the noise stimulus until approximately 20,000 driven spikes were recorded. Due to noise-induced threshold elevation, the SPL of noise stimuli was on average ~20 dB higher in noise-overexposed fibers than in unexposed controls (Figure 2). Noise stimuli were 1.7 s in duration with a bandwidth of 16.5 kHz and silent interval between stimuli of 1.2 s.

### Wiener-kernel computations

*h*_2_ was computed from 2nd-order cross-correlation between the Gaussian noise stimulus waveform *x(t)* and the response train of *N* = ~20,000 driven spikes (Figure 1A). Only spikes occurring more than 20 ms after stimulus onset and before stimulus offset were included in the cross-correlations, which were calculated with a sampling period of 0.02 ms and maximum time lag τ of 10.2 ms (512 points) or 20.4 ms (1024 points; for fibers with CF < 3 kHz). The basic computations for *h*_2_ have been described previously in detail (van Dijk et al., 1994; Lewis et al., 2002; Recio-Spinoso et al., 2005; Temchin et al., 2005) Briefly, *h*_2_(τ_1_,τ_2_) is calculated as $\frac{N_{0}}{2A^{2}}$[*R*_2_(τ_1_, τ_2_) − ϕ_*xx*_(τ_2_ − τ_1_), where τ_1_ and τ_2_ are time lags, *N*_0_ is the mean driven spike rate, *A* is the instantaneous power of the noise, $R_{2}\left(\tau_{1},\;\tau_{2}\right)=\frac{1}{N}\sum_{i=1}^{N}x(t_{i}-\tau_{1})x(t_{i}-\tau_{2})$ is the 2nd-order reverse-correlation function, and ϕ_*xx*_ (τ) is the autocorrelation function of the stimulus. So computed, *h*_2_ is a matrix with units of spikes·*s*^−1^ · *Pa*^−2^ that represents the non-linear interaction or “cross-talk” between the responses to two impulses (Recio-Spinoso et al., 2005).

In practice, *h*_2_ can contain bands running parallel to the diagonal (separated in time by ~1/CF), reflecting phase locking to the temporal envelope of the stimulus, and perpendicular to the diagonal, reflecting non-linearity in the phase-locked response to the temporal fine structure. In its 2-dimensional Fourier transform, the envelope component of *h*_2_ falls into the 2nd and 4th quadrants while the fine structure component falls into quadrants 1 and 3 (Recio-Spinoso et al., 2005). Because our primary focus was envelope coding, we removed the fine structure component from *h*_2_ by discarding the contents of the 1st and 3rd quadrants.

### Quantifying temporal envelope coding

We quantified the temporal precision of envelope coding using spectral and temporal analysis methods. For the spectral analysis, we computed the STRF as the 1-dimensional Fourier transform of *h*_2_ (Figure 1B; Lewis and van Dijk, 2004). Next, the modulation tuning function was calculated at the 2-dimensional Fourier transform of the STRF (Figure 1C), and collapsed across spectral modulation frequencies to yield a TMTF (Figure 1F, black line; as in Woolley et al., 2005). This TMTF describes the temporal profile of the STRF. TMTFs were quantified based on roll-off rate (Figure 1F, green line; measured over the region of the function between peak amplitude and 6 dB below the peak) and cut-off modulation frequencies falling −3, −4, −5, and −6 dB from peak amplitude (red dotted lines). A noise floor for each TMTF (Figure 1F, gray dotted line) was generated from portions of the STRF occurring before and after the response (i.e., the first 2 ms and last 3 ms of the STRF in Figure 1B) using the same computations. Regions of the TMTF rising less than 3 dB above the noise floor were excluded as not statistically significant. In general, slower (less negative) roll-off rates and greater cut-off modulation frequencies correspond to greater temporal precision of envelope coding.

For the temporal analysis, we calculated the first eigenvector of *h*_2_ as in previous work (Figure 1E, black line; Lewis et al., 2002; Recio-Spinoso et al., 2005). The amplitude envelope of this eigenvector (Figure 1E, gray line), determined using the Hilbert transform, represents the temporal window to which the fiber responds to acoustic stimulation with an increase in spike rate. For the fiber shown in Figure 1, for example, spikes are evoked by acoustic energy falling in a temporal window occurring 2.5–5 ms previously. The noise floor of the temporal window was calculated as the mean amplitude plus 3 standard deviations during the first 2 ms and last 3 ms of the waveform. Portions of the temporal window falling below the noise floor were excluded as not statistically significant. We quantified the duration of the temporal window at amplitude values corresponding to 20, 30, 40, and 50% of peak amplitude (Figure 1D, red dotted lines). In general, shorter durations at any given amplitude value reflect greater temporal precision.

Finally, we used the amplitude envelope of *h*_2_ to quantify the amplitude and latency of temporal envelope coding (Figure 1E, black cross). To facilitate comparison of data across fibers with varying driven rates and response threshold levels, we normalized *h*_2_ by dividing by *N*_0_*A*. So normalized, the amplitude values relate to a modulation factor of the mean firing at the average SPL of the noise stimulus.

### Statistical analysis

Statistical analyses were conducted using local regression (LOESS procedure) and mixed models in SAS (MIXED procedure; SAS Institute Inc.). Dependent variables were log_10_-transformed in all cases except for the analysis of temporal window latency. Local regressions were performed with a smoothing parameter of α = 0.5. In general, mixed model analyses were conducted with two continuous independent variables, log_10_(CF) and [log_10_(CF)]^2^. [log_10_(CF)]^2^ was dropped from the model when not statistically significant (*P* > 0.05). Categorical independent variables included hearing status (normal vs. noise-overexposed) and spontaneous rate group (low[≤20 spikes/s] or high). Statistical inferences were drawn based on *F*-tests and *T*-tests comparing least squares means.

## Results

### Pattern of hearing loss

**Figure 3:**
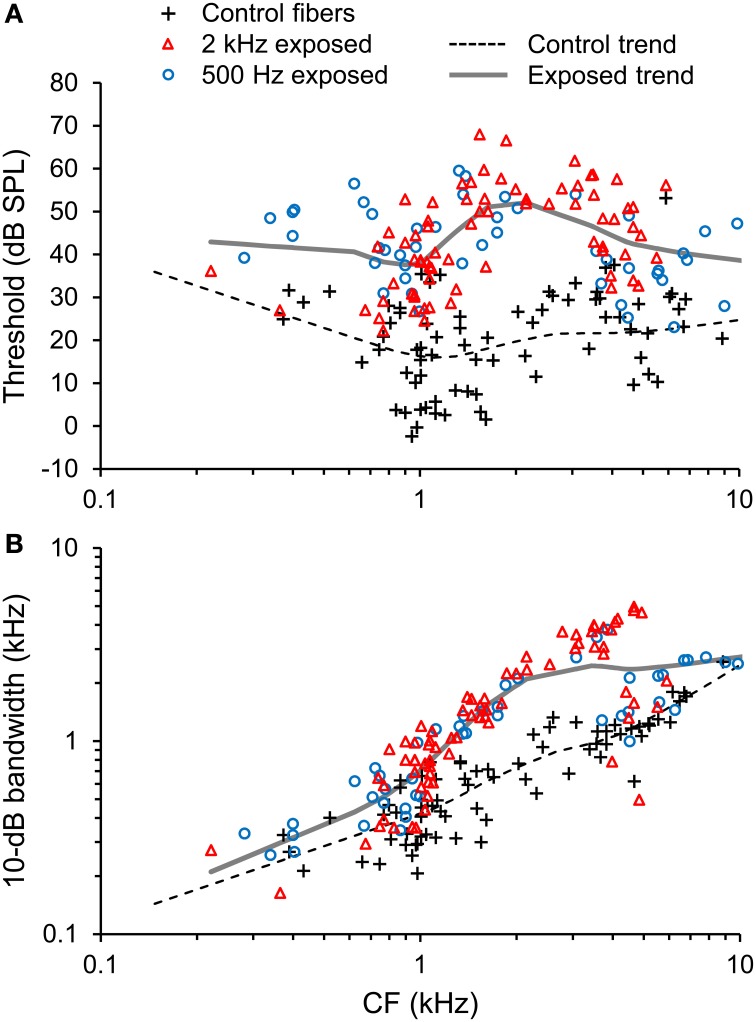
**Pattern of noise-induced sensorineural hearing loss (SNHL). (A)** Tuning curve thresholds and **(B)** and 10-dB bandwidth plotted as a function of CF in normal-hearing control fibers and fibers overexposed to noise. Trend lines are based on local regression analyses. Noise overexposure was associated with threshold elevation and increased tuning bandwidth.

Acoustic overexposure was associated with increases in both the threshold and 10-dB bandwidth of auditory-nerve fiber tuning curves (Figure 3). Threshold elevation and increased tuning bandwidth were most pronounced in fibers with CFs from 1.5 to 4 kHz, but occurred to some extent across the entire CF range studied (0.2–10 kHz).

Patterns of hearing loss were similar between the 2 kHz narrowband and 500 Hz octave band overexposure paradigms (Figure 3, red triangles and blue circles, respectively). For example, mean threshold elevation at CFs of 0.5, 1, 2, 4, and 8 kHz were 8, 20, 33, 25, and 25 dB, respectively, for the 2 kHz exposure and 20, 25, 26, 21, and 16 dB, respectively for the 500 Hz exposure. Temporal response patterns were also similar between the overexposed groups, and are therefore described together.

**Figure 4:**
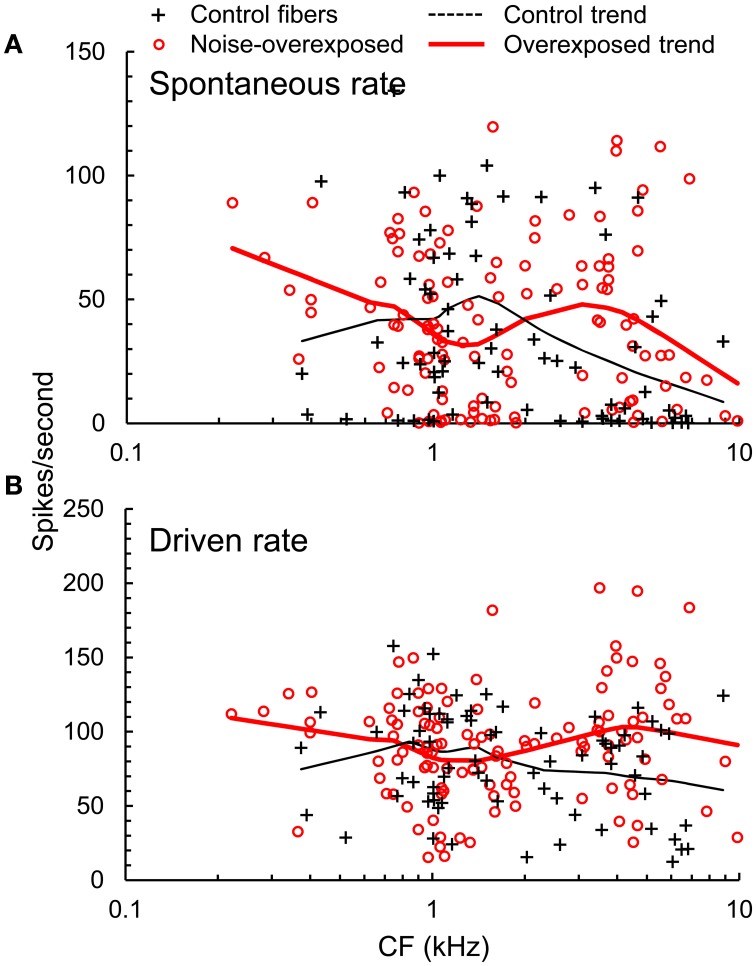
**Firing rates. (A)** Spontaneous rate (SR) and **(B)** driven firing rate plotted as a function of CF in normal hearing and noise-overexposed auditory-nerve fibers. Trend lines are based on local regression analyses. At CFs above 2.5 kHz, noise overexposure was associated with an increase in driven rate and decrease in the proportion of low-SR fibers.

In basal auditory-nerve fibers with CFs > 2.5 kHz, noise overexposure was associated with a decrease in the proportion of fibers with low(≤20 spikes/s) spontaneous rates (SR; Chi-square = 4.79, *DF* = 1; *P* = 0.029; Figure 4A). Furthermore, noise-overexposed basal fibers exhibited an increase in mean driven rate in response to noise stimuli presented at 10–15 dB sensation level [mean difference [±*SE* = 31.9 ± 9.8 spikes/s, *t*_(65)_ = 3.25, *P* = 0.002; Figure 4B], suggestive of an increase in the slope of rate-intensity level functions (for broadband noise). More apical fibers with *CF*s ≤ 2.5 kHz showed no significant variation with noise overexposure in the proportion of low-SR fibers (Chi-square = 0.08, *DF* = 1, *P* = 0.77) or driven firing rate [*t*_(123)_ = −0.06, *P* = 0.95; Figure 4].

### Temporal modulation transfer functions

**Figure 5:**
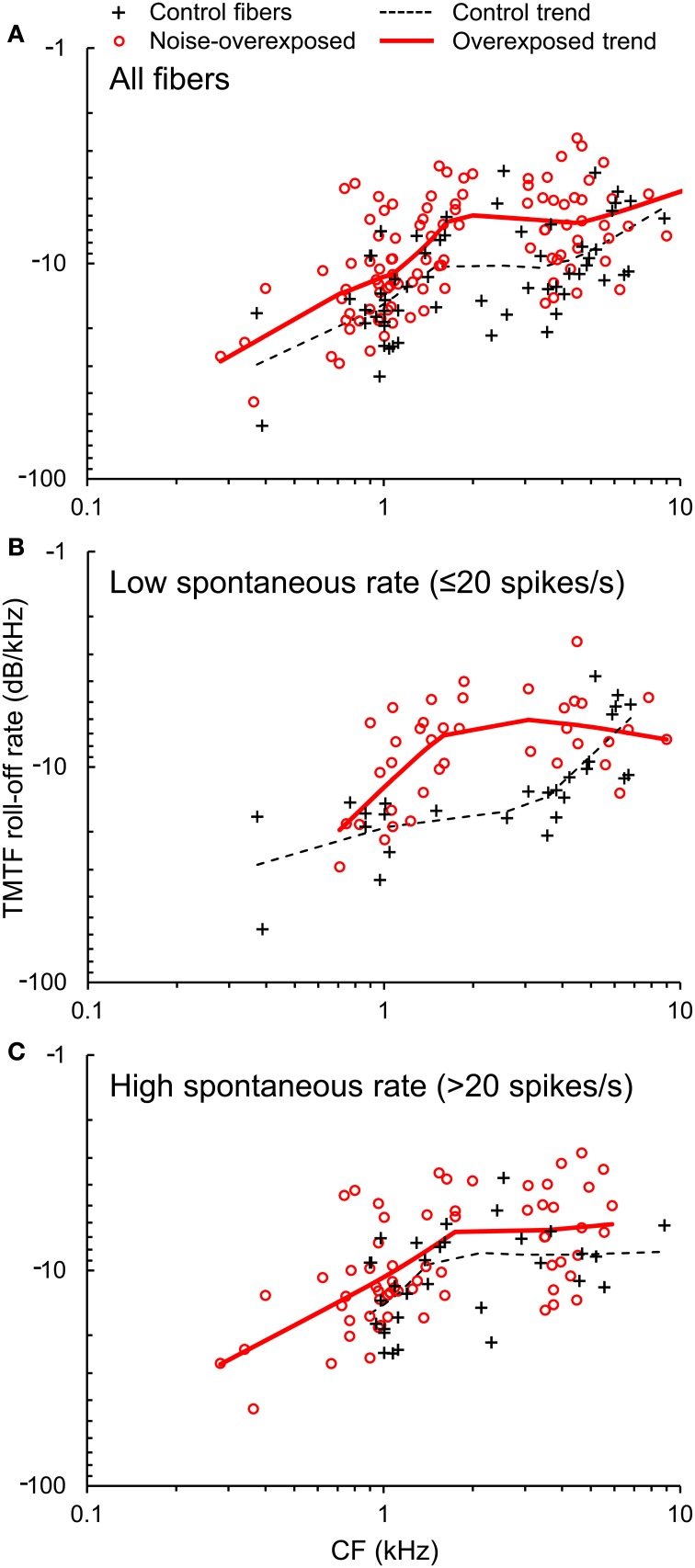
**TMTF roll-off rate. (A)** The roll-off rate of TMTFs plotted as a function of CF in normal hearing and noise-overexposed auditory-nerve fibers. Data from subpopulations of fibers with low and high spontaneous rate are shown in panels **(B)** and **(C)**. Trend lines are based on local regression analyses. Roll-off rate was 30% slower (less negative) in noise-overexposed fibers than in normal-hearing controls.

TMTFs generated from Wiener-kernel analyses of auditory-nerve fiber responses to broadband Gaussian noise invariably exhibited a roll-off in response amplitude with increasing temporal modulation frequency (Figure 1F). In general, roll-off rate was slower (i.e., less negative) in fibers with higher CFs (Figure 5), consistent with expectations for the modulation spectra of cochlear-filtered narrowband noises with larger bandwidths (Figure 3) (Dau et al., 1999). Notably, the roll-off rate was also slower in noise-overexposed fibers than in normal-hearing control fibers. The mean difference in log_10_-transformed roll-off rate ±*SE* was −0.149 ± 0.033 [*t*_(157)_ = −4.45, *P* < 0.001], which corresponds to a 29% reduction in roll-off rate in noise-overexposed fibers compared to normal-hearing controls. This pattern is consistent with increased temporal precision with SNHL.

**Figure 6:**
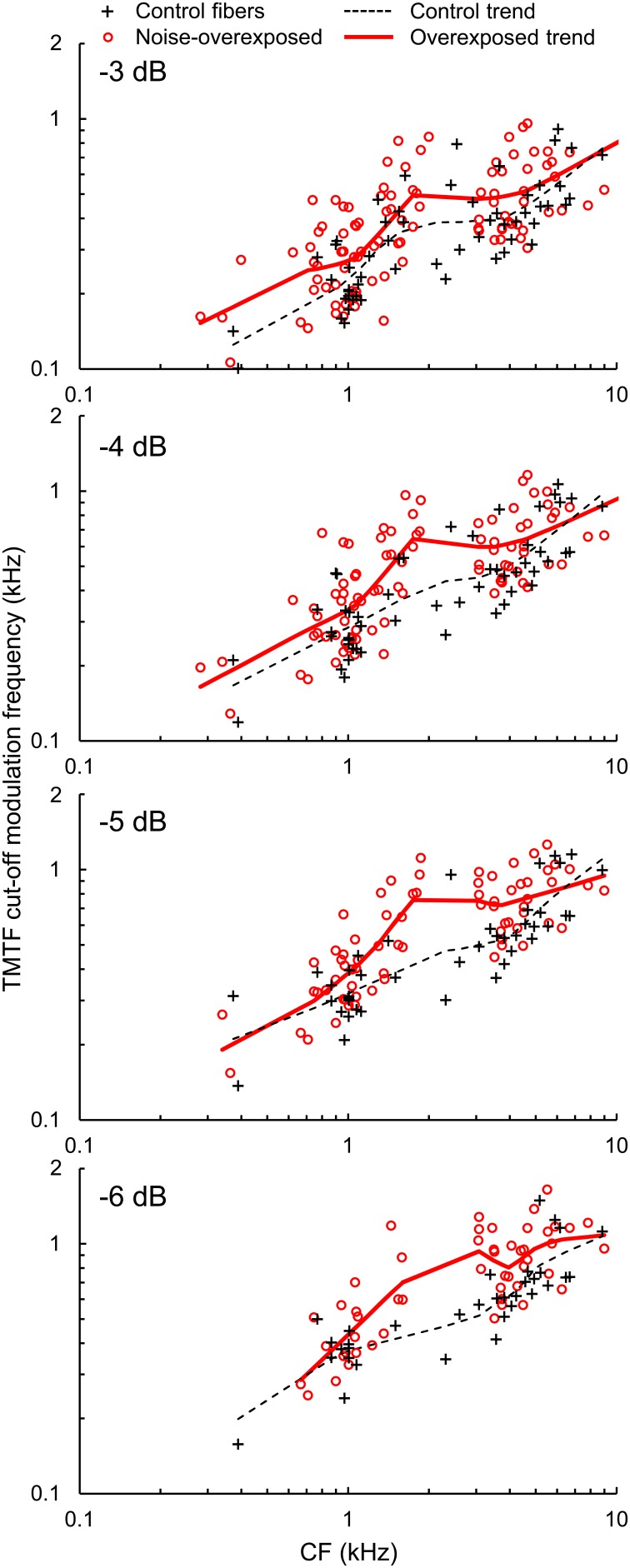
**TMTF cut-off modulation frequencies**. Cut-off modulation frequencies measured 3, 4, 5, and 6 dB down from the peak of the TMTF plotted as a function of CF in normal hearing and noise-overexposed fibers. Modulation cut-offs in dB are marked at the top left of each panel, and trend lines are based on local regression analyses. Cut-off modulation frequencies were 20–25% greater in noise-overexposed fibers than in normal-hearing controls.

Cut-off modulation frequencies were generally greater in noise-overexposed fibers than in normal-hearing control fibers (Figure 6). Mean differences (±*SE*) in log_10_-transformed cut-off modulation frequencies measured 3, 4, 5, and 6 dB down the TMTF were 0.0741 ± 0.0249 [*t*_(154)_ = 2.98, *P* = 0.003], 0.0833 ± 0.02509 [*t*_(136)_ = 3.32, *P* = 0.001], 0.0989 ± 0.0248 [*t*_(110)_ = 3.99, *P* < 0.001], and 0.1036 ± 0.0270 [*t*_(82)_ = 3.84, *P* < 0.001], respectively. These results are consistent with the analysis of roll-off rate, and correspond to a 20–25% increase in cut-off modulations with our noise-induced SNHL.

TMTF parameters were generally similar between subpopulations of auditory-nerve fibers with low and high-SRs (Table 1; Figures 5B,C), except for the −3 dB cut-off modulation frequency, which was moderately greater in the high-SR group (mean difference in log_10_-transformed cut-off modulation frequency [±*SE*]: 0.0532 ± 0.0254; *t*_(152)_ = 2.09, *P* = 0.038).

**Table 1:** **Statistical tests for effects of SR group on temporal precision**.

**Dependent variable**	**Effect**	***F*-value**	***DF***	***P*-value**
TMTF slope	SR	2.90	1,155	0.090
	SR*exposure	1.88	1,155	0.17
TMTF -3 dB cutoff	SR	4.32	1,152	0.038
	SR*exposure	0.62	1,152	0.43
TMTF -4 dB cutoff	SR	1.70	1,134	0.19
	SR*exposure	0.55	1,134	0.46
TMTF -5 dB cutoff	SR	0.01	1,108	0.93
	SR*exposure	0.24	1,108	0.62
TMTF -6 dB cutoff	SR	0.02	1,80	0.89
	SR*exposure	0.03	1,80	0.87
Temporal window duration (50% peak amplitude)	SR	0.60	1,181	0.44
	SR*exposure	0.08	1,181	0.77
Temporal window duration (40% peak amplitude)	SR	0.04	1,179	0.85
	SR*exposure	0.10	1,179	0.75
Temporal window duration (30% peak amplitude)	SR	0.13	1,170	0.72
	SR*exposure	0.44	1,170	0.51
Temporal window duration (20% peak amplitude)	SR	0.31	1,151	0.58
	SR*exposure	2.04	1,151	0.16

*DF lists the numerator followed by the denominator degrees of freedom*.

### Temporal windows

**Figure 7:**
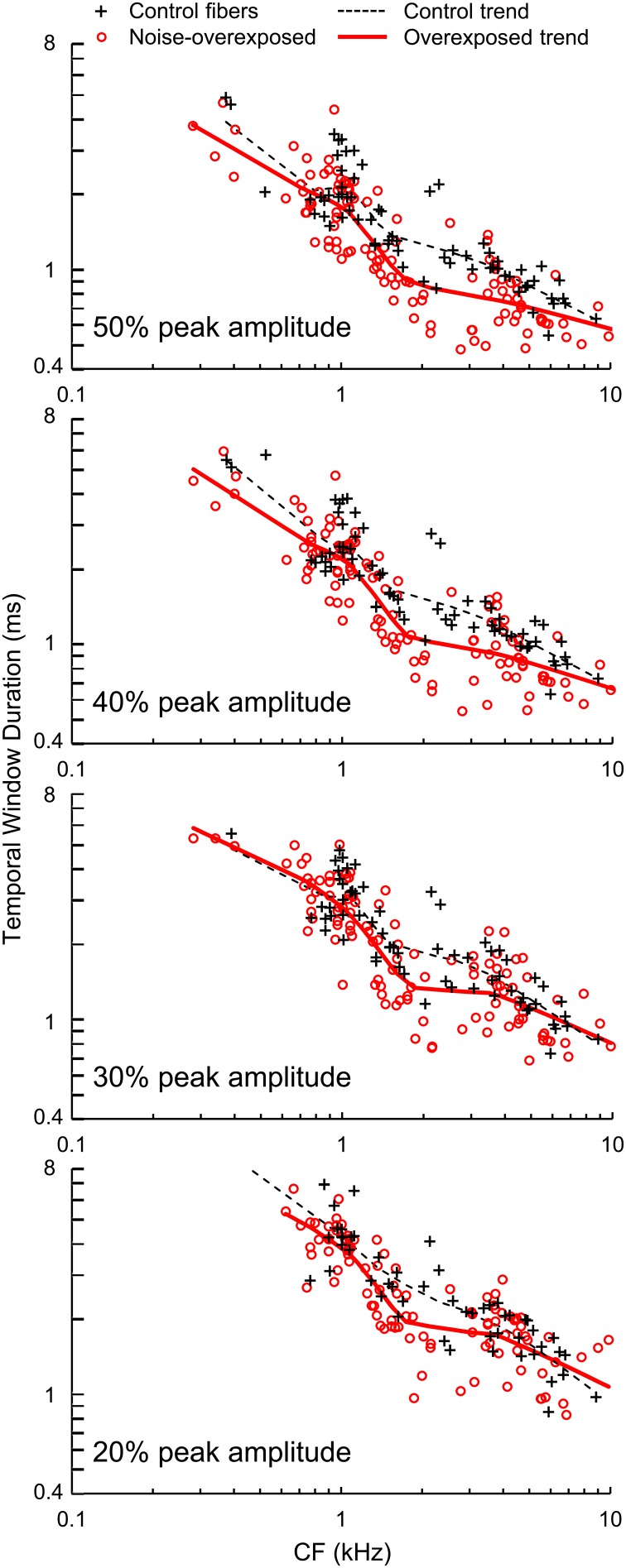
**Temporal window duration**. Durations of temporal windows measured at 50, 40, 30, and 20% of peak amplitude plotted as a function of CF in normal hearing and noise-overexposed auditory-nerve fibers. Percent of peak amplitude is marked at the bottom left of each panel, and trend lines are based on local regression analyses. Temporal windows were 10–20% shorter in noise-overexposed fibers than in normal-hearing controls.

Temporal windows of auditory-nerve fiber sensitivity to acoustic stimulation decreased in duration with increasing CF and moreover, were shorter in noise-overexposed fibers than in normal-hearing controls (Figure 7). Mean differences (±*SE*) in log_10_-transformed window duration measured at 50, 40, 30, and 20% of peak amplitude were −0.0982 ± 0.0189 [*t*_(1830)_ = −5.20, *P* < 0.001], −0.0915 ± 0.0185 [*t*_(181)_ = −4.94, *P* < 0.001], −0.0568 ± 0.0199 [*t*_(172)_ = −2.86, *P* = 0.005], and −0.0537 ± 0.0204 [*t*_(153)_ = −2.63, *P* = 0.009], respectively, consistent with the TMTF analyses. On average, noise-induced SNHL decreased the duration of temporal sensitivity by 10–20%, with greater reductions observed for measurements taken closer to peak amplitude (i.e., 40 and 50% of peak amplitude).

Temporal window duration and changes in temporal window duration with noise overexposure were similar between fibers with low and high SRs (Table 1).

### Amplitude and latency of temporal envelope coding

**Figure 8:**
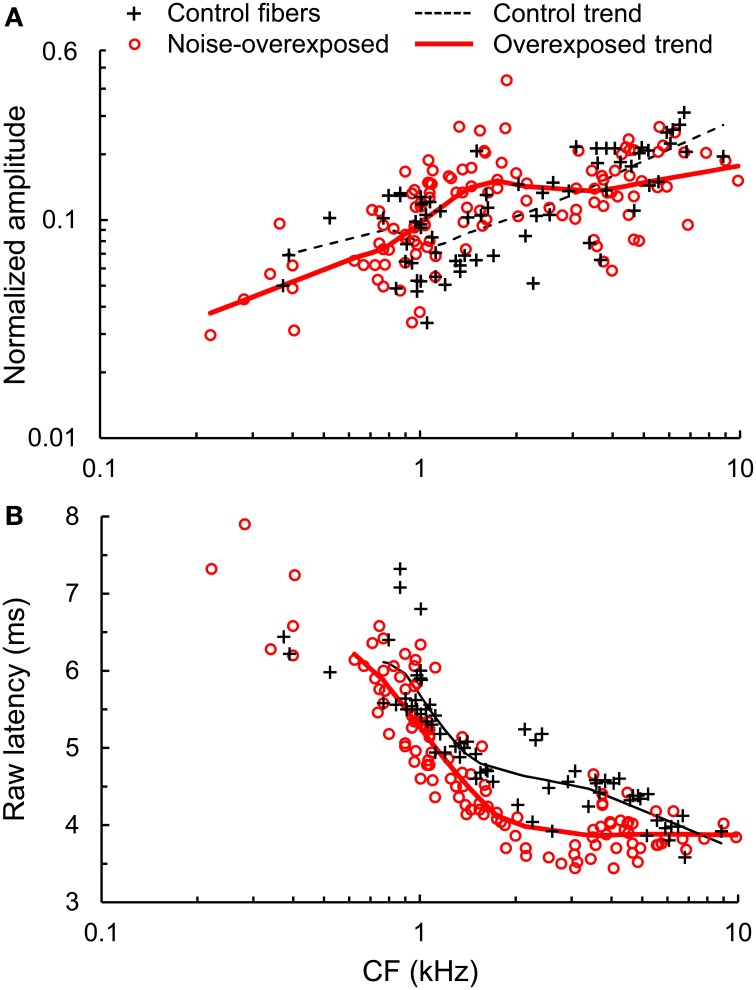
**Amplitude and latency of temporal envelope coding. (A)** Normalized amplitude and **(B)** raw latency of temporal envelope coding plotted as a function of CF in normal hearing and noise-overexposed auditory-nerve fibers. Trend lines are based on local regression analyses. Latency values include a system delay in addition to neural response latency. Noise overexposure was associated with amplified envelope coding in fibers with CFs from 1 to 2 kHz (~50% amplification) and shorter response latency (by ~0.4 ms) across all CFs.

Noise-induced SNHL was associated with a moderate increase in the amplitude of envelope coding in fibers with CFs between 1 and 2 kHz (Figure 8A). The mean (±*SE*) increase in log_10_-transformed amplitude was 0.193 ± 0.044 [*t*_(60)_ = 4.38, *P* < 0.001], which corresponds to an increase of 56%. Envelope coding at higher and lower CFs was similar between noise-overexposed fibers and normal-hearing controls [*CF* > 2: *t*_(73)_ = −1.91, *P* = 0.06; *CF* < 1: *t*_(46)_ = −0.57, *P* = 0.57].

The latency of envelope coding decreased with increasing CF and notably, was shorter in noise-overexposed fibers than in normal-hearing controls (Figure 8B). Across fibers with CFs greater than 0.6 kHz, noise-induced SNHL decreased latency by 0.434 ± 0.061 ms [mean ± *SE*; *t*_(174)_ = −7.08, *P* < 0.001].

## Discussion

The results of the present study show that noise-induced SNHL increases the temporal precision of envelope coding in auditory nerve fibers by 20–30% at equal stimulus sensation level (i.e., 10–15 dB above threshold). Furthermore, SNHL decreases response latency by 0.4 ms and, in fibers with CFs from 1 to 2 kHz, amplifies the representation of envelope structure by 50%.

The increase in temporal precision with SNHL demonstrated here can most likely be attributed to broader cochlear frequency tuning. Broadly tuned systems have a short impulse response that increases sensitivity to rapid temporal envelope modulations of the input stimulus. Interestingly, tuning curve bandwidths of some noise-overexposed fibers were 100–200% greater than in normal-hearing controls, but temporal precision rarely increased by more than 20–30%. This difference suggests that while broader frequency tuning with SNHL may allow an increase in the temporal precision of envelope coding, temporal precision can only increase to a degree before it becomes constrained by additional limiting factors such as neural refractoriness and adaptation. A similar phenomenon appears to occur in the normal-hearing cochlea of cats, where temporal precision increases with increasing tuning bandwidth up to a CF of approximately 10 kHz, above which temporal precision remains constant despite further increases in tuning bandwidth (Joris and Yin, 1992).

Kale and Heinz (2012) examined the effects of SNHL on the temporal precision of envelope coding using TMTFs generated from auditory-nerve fiber responses to SAM tones. While -3-dB cut-off modulation frequency showed similar CF dependence between SAM tone-based TMTFs and TMTFs from the current study, SAM tone-based TMTFs failed to show a consistent change in temporal precision with SNHL. Wiener kernel-based TMTFs may be more sensitive to the effects of SNHL because, due to the stimulus, fibers are stimulated over their entire frequency tuning bandwidth with a dynamically varying temporal envelope. This task may not only be more challenging, but also more representative of the fiber's behavior during processing of perceptually relevant signals such as speech in fluctuating background noise.

While our results show that temporal precision increases with noise-induced SNHL at equal sensation level, it remains unclear how temporal precision might compare between normal hearing and noise-overexposed auditory-nerve fibers at equal SPL. If temporal precision in the normal-hearing cochlea increases with SPL, as might be expected based on increasing tuning bandwidth with level, temporal precision might converge somewhat between groups at equal SPL. Note, however, that outer hair cell dysfunction causes broader-than-normal frequency tuning up to at least 75 dB SPL (Ruggero and Rich, 1991), suggesting that increased temporal precision with SNHL should persist. Furthermore, previous studies using SAM tone stimuli have shown relatively limited variation in TMTF shape with stimulus level in normal-hearing animals (Joris and Yin, 1992). Greater knowledge of changes in temporal precision with sound level in both normal hearing and impaired cochleae are high priorities for future research.

It should be noted that CF in noise-overexposed fibers was assigned based on the breakpoint in the high-frequency slope of the tuning curve (Liberman, 1984). While we have no reason to suspect that estimates of CF were biased in this group, significant overestimation would be expected to result in a finding of enhanced temporal precision because temporal precision increases with increasing CF (e.g., see Figures 5–7).

The decrease observed in the proportion of basal auditory-nerve fibers with low SRs is consistent with previous findings that noise overexposure causes selective degeneration of low-SR fibers. Underrepresentation of low-SR fibers was noted in an earlier study of noise-induced permanent hearing loss in cats (Liberman, 1978). More recent results from a study of guinea pigs suggest that even temporary threshold shifts associated with mild noise overexposure lead to selective degeneration of low-SR fibers (Furman et al., 2013).

While selective loss of low-SR fibers due to SNHL is expected to adversely affect perceptual abilities under real-world listening conditions based on particularly robust coding of signals in noise and high-SPL signals in this group (e.g., Costalupes et al., 1984), underrepresentation of low-SR fibers did not appear to contribute to the differences in temporal precision observed in the present study (see Table 1). Differences in temporal precision related to SR might be more prominent at higher SPL. Differences in temporal precision were also not obviously related to mean driven rate. Whereas increases in driven rate were limited to fibers with CFs above 2.5 kHz, changes in temporal precision spanned the entire CF range sampled.

Our finding of amplified envelope coding is consistent with the results of previous physiological work involving SAM tones and single-formant stimuli (Kale and Heinz, 2010). Amplified envelope coding with SNHL may be related to a variety of factors including a reduction in fast-acting cochlear compression with outer hair cell damage. Reduced compression, which has been hypothesized to underlie perceptual “loudness recruitment” or abnormal growth of loudness with increasing SPL, increases the slope of the input-output function of the basilar membrane and therefore leads to larger modulations of basilar membrane velocity (and hence, spike rate) for a given modulation of the stimulus amplitude envelope. Amplified envelope coding with SNHL may also reflect increases in the slope of auditory-nerve fiber rate level functions associated with partial inner hair cell damage (loss of component-1 due to loss of the tallest row of stereocilia; Liberman and Kiang, 1984; Heinz and Young, 2004; Kale and Heinz, 2010; but see Figure 4) and changes in auditory-nerve response temporal dynamics (Scheidt et al., 2010).

Taken together with other physiological data (Kale and Heinz, 2010, 2012), these new results help explain previous behavioral findings of enhanced perceptual salience of envelope structure with SNHL. In individuals with unilateral SNHL, more modulation depth must be applied to 1-kHz tones presented to the unimpaired ear than the impaired ear to evoke a sensation of equal modulation depth (Moore et al., 1996).

The 0.4 ms decrease in response latency with noise-induced SNHL found here is consistent with previous results showing reduced response latency to clicks and tone bursts with SNHL, at least at equal sensation level. Noise-overexposure in chinchillas decreases the latency of auditory-nerve fiber onset responses to clicks and tones (Salvi et al., 1979; Scheidt et al., 2010), while kanamycin-induced damage in guinea pigs decreases compound action potential latency (Wang and Dallos, 1972). Similarly, studies employing scalp-recorded auditory evoked potentials have demonstrated reductions in response latency in chinchillas (Henry et al., 2011) and human subjects (Don et al., 1998; Strelcyk et al., 2009). The differences in response latency at equal (high) SPL is not known, but might be smaller in magnitude based on previous findings that the latency of second-order Wiener kernels decreases with increasing level in normal-hearing chinchilla auditory-nerve fibers (Recio-Spinoso et al., 2005).

In conclusion, the changes in envelope coding demonstrated here may contribute to speech perception problems in people with SNHL. Stronger coding of temporal envelope cues and coding of faster envelope modulations may serve as distractions from more relevant cues needed to perceive speech in environments with fluctuating background noise. Several studies have simulated loudness recruitment in normal-hearing listeners to examine the possible effects of enhanced temporal envelope structure on perception of speech in noise. Enhanced envelope structure was shown to increase speech discrimination thresholds by up to 6 dB in steady background noise and by 11–13 dB in single-talker babble (Moore and Glasberg, 1993; Moore et al., 1995). The development of new speech processing strategies aimed at restoring normal-hearing temporal envelope coding in the impaired cochlea may be a promising direction for future research.

## Author contributions

Kenneth S. Henry, Sushrut Kale, and Michael G. Heinz designed the experiments and collected data. Kenneth S. Henry analyzed the data and wrote the manuscript with assistance from Sushrut Kale and Michael G. Heinz.

### Conflict of interest statement

The authors declare that the research was conducted in the absence of any commercial or financial relationships that could be construed as a potential conflict of interest.
